# Perception of Parental Attitudes and Self-Efficacy in Refusing Alcohol Drinking and Smoking by Spanish Adolescents: A Cross-Sectional Study

**DOI:** 10.3390/ijerph20010808

**Published:** 2023-01-01

**Authors:** José Jesús Gázquez Linares, Ana Belén Barragán Martín, María del Mar Molero Jurado, María del Mar Simón Márquez, María del Carmen Pérez-Fuentes, África Martos Martínez, Rosa María Del Pino Salvador

**Affiliations:** 1 Department of Psychology, University of Almería, 04120 Almería, Spain; 2 Department of Psychology, Universidad Autónoma de Chile, Providencia 7500912, Chile

**Keywords:** parental attitudes, self-efficacy, adolescents, alcohol use, smoking, tobacco

## Abstract

Adolescents are particularly vulnerable to initiation of the use of substances harmful to health, and its increase is cause for concern. The objective of this study was to analyze the relationship between self-efficacy in refusing alcohol and the attitude of adolescents toward drugs and their perception of their parents’ attitude toward refusal. The study was carried out in 2019 in a sample of 1287 students from 11 public high schools in the province of Almería (Spain). Students were aged 14 to 18 in their 3rd and 4th year of compulsory secondary education. The Drinking Refusal Self-Efficacy Questionnaire—Revised Adolescent version (DRSEQ-RA), Attitudes Toward Taking Drugs—Basic BIP Scale and the Parents’ attitudes Toward Drug Use were administered. The results showed that family relationships seem to have a direct impact on adolescent patterns in smoking and drinking alcohol. However, a favorable attitude toward drugs is a risk factor for drinking alcohol and smoking tobacco. The self-efficacy dimension also acts as a protective factor against the probability of using alcohol or tobacco. The conclusions emphasized that communication within the family core can increase or decrease the risk of adolescents using substances harmful to health.

## 1. Introduction

Adolescence may be considered the stage of human development most vulnerable to the use of substances harmful to health. This is the consequence of a diversity of interacting biological, cognitive, affective and social transformations during that stage [1,2], which is therefore marked by significant changes [3,4,5]; modular development, including functional segregation and fusion with the various brain connections involved, must also be taken into account [6]. During adolescence, the most important changes in brain development occur in the frontal lobe and limbic system. The frontal lobe is associated with the self-regulation that makes an individual rational, and the limbic system is related to generating emotions. The amygdala and hippocampus, both in the limbic system, are related to impulsivity and instinctive emotions. They are activated when the frontal lobe is not yet completely developed. Therefore, adolescents whose frontal lobe is still incomplete are usually more impulsive than rational. As a result, many teenagers act impulsively and emotionally and may experience psychological problems such as addiction and use of alcohol [7], as well as development of specific characteristics, such as impulsive personality traits [8,9]. This development is connected to such factors as environment, genetics and lifestyle [10,11,12,13].

### 1.1. Use of Legal and Illegal Drugs in Adolescence

At present, the use of substances such as alcohol is a frequent generalized practice in adolescents, which negatively impacts their health [14,15,16,17] and psychological and social development [18,19,20,21]. Alcohol has the highest percentage of use, followed by tobacco and cannabis [22,23].

Adolescent use of harmful substances has been increasing yearly, and initiation of its use begins at an increasingly earlier age, when sensation-seeking becomes more evident [24,25]. Young people may also be subjected to stressful social pressures directly related with the use of addictive substances [26]. Similarly, certain dangerous behaviors such as drinking alcohol, smoking tobacco or taking illegal drugs commonly begin in adolescence leading to injuries, violent behaviors, risky sexual behavior, low academic performance, development of mental illnesses, and, in some cases, later dependence [27,28,29,30]. Moral detachment, violent behaviors and a tendency to seek trouble may also appear [18,19,20,21]. Therefore, it is important for the start and continuation of such behavior to be dealt with while its prevention is still possible [27], especially considering that the attitudes of young people toward substance use are related to the use itself, as excessive substance use is linked to a favorable adolescent attitude toward it [31].

Several factors influence the pattern of drinking and smoking in adolescence [32], including heredity, social context, family or peer group dynamics [33]. The crucial influence of the latter has been widely discussed in the literature [34,35,36] and established as one of the key reference groups during this stage [37]. However, the family setting is also critical in influencing substance use in youths. Adolescents who keep up good communication with their families and receive the message that drinking alcohol is dangerous for health show a pattern of later initial use and lower consumption frequency [20,21,38]. Along this line, parental disapproval of substance use and the presence of a confidante to whom they can tell that their child is using a substance have both been related to a lower probability of drinking alcohol and smoking during adolescence [39]. On the contrary, when there are frequent conflicts in the family core and poor communication among its members, the parents drink and/or they are permissive, the risk of adolescent drug use increases [40]. Studies such as the one conducted by Cerezo et al. [41] have shown that two family factors, indifference to substance use and a tolerant and permissive parenting style, are moderators increasing adolescent use.

Fascination with the dangerous situation of drinking alcohol is also a determining factor along with the influence of their social environment in the response to starting, and later continuing, the excessive use of alcohol [42]. Qualitative studies such as the one conducted by Jacob et al. [32] suggest that some parents consider experimenting with alcohol inevitable, and that part of that requires the adolescent to experience drinking too much alcohol. Similarly, a good parent–child relationship in which psychological flexibility is prioritized entails a positive parenting style [43,44] where there is open, quality communication with the children, understood to be essential for them to reduce such behavioral attitudes [45,46,47,48]. Smoking tobacco is similar. Formation of a smoking habit is reinforced when one or both of the parents are smokers. Exposure to certain environments where it is normalized, such as publicity in communication media, also exerts a strong influence [49,50,51,52,53]. Therefore, the relationship with their parents and their parents’ attitudes must be kept in mind when studying adolescents and their substance use. This study dealt with that relationship.

### 1.2. Self-Efficacy in Refusing Legal and Illegal Drugs

According to Olivari and Barra [54], self-efficacy is related to vulnerability to engaging in negative behavior such as substance use and later abuse, because it is related to information processing in different contexts [55]. In this regard, self-efficacy in refusing to drink refers to the confidence that individuals place in their own ability to resist drinking alcohol [56].

Risk behaviors such as drinking alcohol and smoking may begin as ways to facilitate adaptation by joining a peer group [57]. Therefore, when adolescents expect substance use to help them fit in with other youths, they are reinforcing the perception and memorizing that the consequences of harmful substance use are those desired: by drinking and/or smoking, they can join a group of peers [58]. High expectations for social benefits increase risk of later use, and believing that these behaviors have positive effects considerably weakens the adolescents’ self-efficacy in refusing these drugs [56,59]. On the contrary, high self-efficacy, along with health education, are related to a healthy lifestyle [60]. Examining the attitudes and self-efficacy of youths in opposing use is crucial for prevention and early intervention. Lima-Serrano et al. [61], for example, showed that self-efficacy in refusing to smoke is one of the strongest variables associated with the intention of not smoking in youths, while the perceived use pattern around them is more relevant in their behavior (that is, not smoking). The intention to drink and drinking behavior have been associated negatively with self-efficacy in refusing it [62,63]. However, this variable needs more study to understand its role in refusing alcohol and its relation to other aspects, since in studies such as the one conducted by Dalgaard [64], where intervention with augmented reality was given for this variable, there were no significant effects on preventing drinking.

### 1.3. Objective

The aim of our study was to analyze the relationship between self-efficacy in refusing alcohol and smoking and attitude toward legal and illegal drugs of both the adolescents themselves and their perception of their parents’ attitudes.

### 1.4. Hypotheses

**H1.**  *Unfavorable attitude of parents toward drugs is correlated positively with an unfavorable attitude of adolescents toward drugs*.

**H2.**  *Favorable adolescent attitudes toward drugs correlate negatively with their assessment of their relationship with their parents*.

**H3.**  *Frequency of smoking tobacco and drinking alcohol correlates positively with favorable adolescent attitudes toward drugs and negatively with concern of parents about drugs*.

**H4.**  *Girls score higher on parental concern about drug use, while boys score higher on favorable attitudes toward drug use*.

**H5.**  *The risk of drinking alcohol and smoking tobacco is higher when adolescents’ attitude toward drug use is favorable*.

**H6.**  *Parental concern for drug use is a protective factor against drinking alcohol and smoking tobacco*.

**H7.**  *Perceived self-efficacy in refusing to drink alcohol in the face of social pressure is a protective factor against drinking alcohol and smoking tobacco*.

## 2. Materials and Methods

### 2.1. Study Design and Setting

This study had a descriptive cross-sectional design. Along with sociodemographic data, the variables evaluated were: smoking tobacco (understood as smoking cigarettes) and its frequency; drinking alcohol (which refers to alcoholic drinks) and its frequency; attitude toward drug use (referring to a favorable or unfavorable position toward use of legal and illegal substances); self-efficacy in refusing to drink alcohol (understood as the ability to resist drinking alcohol in different situations); relationship with parents (referring to their treatment and the feelings of adolescents regarding unity with their father and mother or guardian ); and beliefs about the attitude of their parents toward use (understood as the adolescents’ own evaluation of whether their parents support legal or illegal drug use).

### 2.2. Participants

The study sample comprised of a total of 1287 students from 11 public high schools in the province of Almería (Spain). Of the original sample of 1317 students, 30 were discarded because of incongruent, random or incomplete answers. Students were aged 14 to 18 with a mean age of 15.11 (SD = 0.91). The gender distribution was 47.1% (n = 606) boys and 52.9% (n = 681) girls, with a mean age of 15.12 (SD = 0.94) and 15.10 (SD = 0.88), respectively. Distribution by grade level was 55% (n = 707) in 3rd year of high school, and 45% (n = 577) in 4th year. The population is made up of 15,019 students (confidence level of 95% and estimation error of 3%), so a sample of 997 secondary school students (3rd and 4th ESO) was estimated (Table 1).

### 2.3. Measures

An ad hoc questionnaire was prepared to collect sociodemographic data (age, gender, grade level), and included questions related to smoking tobacco and frequency (Do you smoke? How often?), questions about drinking alcohol and frequency (Do you drink alcohol? How often? The answers were rated on an eight-point Likert-type scale (from 0 = “I have smoked very little in my life” up to 7 = “Quite a lot more than a pack a day”). Lastly, questions related to the adolescents’ evaluation of their relationship with their father, mother or guardian (Evaluate your current relationship with your father/guardian; Evaluate your current relationship with your mother/guardian, tated on a five-point Likert scale (from 0 = very bad to 4 = very good).

The Drinking Refusal Self-Efficacy Questionnaire—Revised Adolescent version (DRSEQ-RA) by Patton et al. [56] consists of 19 items that measure drinking refusal self-efficacy. The answers are rated on a six-point Likert-type scale (from 1 = I am very sure that I could not resist drinking, to 6 = I am very sure that I could resist drinking). The questionnaire is made up of three subscales: social pressure refusal self-efficacy (You take the drink offered you and pretend to drink it), opportunistic self-efficacy refusal (You feel you have to drink because you have a hard time saying NO), emotional relief refusal self-efficacy (You try to stay away from the person who is serving the drinks). This questionnaire has been used with Spanish adolescents [65]. The reliability of each of the scales was α = 0.88, α = 0.95, y α = 0.95, respectively.

The Drug Use Attitudes BIP Baseline Scale [66] is comprised of 12 items which evaluate adolescent attitudes and opinions about drug use. Answers are rated on a 6-point Likert-type scale (from completely agree to completely disagree). The instrument’s reliability was α = 0.79.

The Communities that Care Youth Survey [67] was used, selecting one of the risk factors that it evaluates specifically, Parental Attitudes Toward Drug Use. This instrument has been used in samples of Spanish-speaking adolescents [68]. It is made up of three items that measure parental concern with drug use. The answer choices follow a 4-point Likert-type scale (from 1 = Not bad to 4 = Very bad). The instrument’s reliability was α = 0.55.

### 2.4. Procedure

First, before data were collected, a preliminary meeting was held at the school to inform the study participants about the study objectives and guarantee confidential treatment of data. Of the 15 schools selected at random by conglomerates (Center, North, West Side and East Side of the Province of Almeria), 11 agreed to participate and received an individual report with the data corresponding to their school. The data were collected during the first semester of 2019. Once the schedule had been arranged, the questionnaires were administered at the schools in the students’ usual classrooms in the presence of their teacher or tutor. The session started with clear and detailed instructions for the students, who were allocated the time to ask questions and assured that the anonymity of their answers would be retained, therefore ensuring privacy in statistical treatment of data. The students filled in the tests anonymously and individually, taking an estimated time of 15 min to do so. Only those students for whom the school had received parental authorization participated. Parents/guardians provided their consent individually and in writing. In all cases, ethical standards of research were complied with in an informed consent sheet. The study was approved by the University of Almería Bioethics Committee (Ref: UALBIO2018/015).

### 2.5. Data Analysis

First, to explore the relationship between the variables, the bivariate correlation matrix was calculated with the Pearson’s coefficient. Then, the Student’s independent sample t-test was performed to compare groups by gender, whether or not they drank alcohol and whether or not they smoked in relation to self-efficacy in refusing alcohol, parents’ attitude toward legal and illegal drugs and attitudes toward use by adolescents. The effect size was measured using Cohen’s d [69].

Then, binary logistic regression analyses were performed using the enter method. For this, the dependent variables were drinking alcohol and smoking, answered dichotomously (no, yes). The predictor variables in both models were: favorable attitude of adolescents toward drugs, concern of parents about drug use, and self-efficacy in rejecting alcohol (under social pressure, provided opportunity and for emotional relief).

Data were analyzed using the SPSS statistical package v 23 for Windows [70].

In addition, in order to identify the behavior of the sex variable as a moderator of the relationship established between tobacco/alcohol consumption and favorable attitudes toward drugs, a simple moderation analysis was carried out for each case. For this purpose, the PROCESS macro was used to compute models of simple moderation effects [71]. The bootstrapping technique was applied with coefficients estimated from 5000 bootstrap samples.

## 3. Results

### 3.1. Correlation Analysis of Variables

As shown in Table 2, a favorable attitude toward drugs correlated negatively with the three types of self-efficacy: social pressure refusal self-efficacy (*r* = −0.333, *p* < 0.001), opportunistic refusal self-efficacy (*r* = −0.254, *p* < 0.001), and emotional relief refusal self-efficacy (*r* = −0.294, *p* < 0.001).

Parental concern about drug use correlated positively with the three types of self-efficacy in refusing alcohol: social pressure refusal self-efficacy (*r* = 0.199, *p* < 0.001), opportunistic refusal self-efficacy (*r* = 0.164, *p* < 0.001), emotional relief refusal self-efficacy (*r* = 0.171, *p* < 0.001).

There was a negative correlation between social pressure refusal self-efficacy and frequency drinking alcohol (*r* = −0.170, *p* < 0.001) and negative correlation between emotional relief refusal self-efficacy and frequency drinking alcohol (*r* = −0.091, *p* < 0.001).

However, a negative correlation was observed between the adolescents’ assessment of their relationship with both their father (*r* = −0.066, *p* < 0.05) and mother (*r* = −0.109, *p* < 0.001) and favorable attitudes toward drugs, legal and illegal.

However, the correlation between adolescents’ assessment of their relationship with their mother (*r* = 0.066, *p* < 0,05) and parental concern about drug use was positive.

Parental concern about drug use correlated negatively with a favorable attitude toward drugs, legal and illegal (*r* = −0.278, *p* < 0.001).

As shown in Table 2 on the relationships between the parents’ attitude toward legal and illegal drugs and the frequency of drinking alcohol and smoking tobacco, both the frequency of drinking alcohol (*r* = −0.133, *p* < 0.001) and smoking frequency (*r* = −0.108, *p* < 0.001) were negatively correlated with parental concern about drug use.

It also shows that a favorable adolescent attitude toward legal and illegal drugs was positively correlated with the frequency of drinking alcohol (*r* = 0.215, *p* < 0.001) and smoking tobacco (*r* = 0.095, *p* < 0.01).

### 3.2. Self-Efficacy in Refusing Alcohol, Parents’ Attitude toward Drugs and Favorable Attitude toward Drugs and Consumption of Alcohol

Table 3 shows the significant differences in social pressure refusal self-efficacy (*t =* 2.34; *p* < 0.05), opportunistic refusal self-efficacy (*t =* −5.51; *p =* 0.000), and emotional relief refusal self-efficacy (*t =* −4.25; *p <* 0.001) by gender. Boys scored slightly higher in social pressure refusal self-efficacy (*M =* 22.33; *SD =* 6.93) than girls did (*M =* 21.43; *SD =* 6.91). In opportunistic refusal self-efficacy, it was girls who scored higher (*M =* 38.93; *DT =* 7.49) than boys did (*M =* 36.25; *SD =* 9.62). Girls also scored higher in emotional relief refusal self-efficacy (*M =* 37.52; *SD =* 8.07) than boys did (*M =* 35.39; *SD =* 9.71).

Furthermore, there were significant differences between parental concern about drug use (*t = −*2.66; *p* < 0.01) by gender. As observed, girls scored higher on parental concern about drug use (*M =* 3.64; *SD =* 0.43) than boys did (*M =* 3.57; *SD =* 0.49). Finally, there were significant differences between the attitudes toward use (*t = −*3.67; *p* < 0.001) by gender, where boys had a higher mean score in attitudes toward use (*M =* 28.29; *SD =* 8.74) than girls did (*M =* 26.58; *SD =* 7.90).

Table 4 shows the significant differences for social pressure refusal self-efficacy (*t =* 19.99; *p* < 0.001), for opportunistic refusal self-efficacy (*t =* −3.65; *p* < 0.001), and for emotional relief refusal self-efficacy (*t =* 6.37; *p* < 0.001) in drinking alcohol or not. Thus, those who did not drink alcohol had a higher mean score (*M =* 25.34; *SD =* 5.87) in social pressure refusal self-efficacy than those who did drink (*M =* 18.58; *SD =* 6.23). Those who did not drink alcohol had a higher mean score (*M =* 38.57; *SD =* 7.95) in opportunistic refusal self-efficacy than those who did (*M =* 36.81; *SD =* 9.22). Finally, those who said they did not drink had a higher mean score (*M =* 38.13; *SD =* 7.93) in emotional relief refusal self-efficacy than those who said they did drink alcohol (*M =* 35.00; *SD =* 9.56).

In another area, parents’ attitudes toward legal and illegal drugs (*t =* 7.52; *p* < 0.001) differed significantly depending on whether or not they drank alcohol. Those who said they did not drink alcohol scored higher on parents’ attitude toward drugs (*M =* 3.70; *SD =* 0.43) than those who drank (*M =* 3.51; *SD =* 0.47).

There were also significant differences in attitude toward use (*t =* −10.83 ***; *p* < 0.001) depending on whether or not they drank alcohol. Thus, those who said they drank had a higher score in favorable attitude toward drug use (*M =* 24.89; *SD =* 7.32) than those who did not drink alcohol (*M =* 24.89; *SD =* 7.32).

### 3.3. Parents’ Attitude toward Drugs. Favorable Attitude toward Drugs Depending on Adolescents and Belief about Whether Their Parents Would Allow Them to Drink Alcohol and Smoke Tobacco

Table 5 shows the significant differences in parents’ attitude toward drugs (*t =* 4.784; *p* < 0.001) depending on whether they smoked or not. Thus, those who said they did not drink alcohol scored higher on parental concern for use (*M =* 3.63; *SD =* 0.45) than those who did (*M =* 3.46; *SD =* 0.49).

Favorable attitude toward drugs and smoking tobacco showed significant differences (*t =* −10.49; *p* < 0.001) between those who said they smoked (*M =* 33.21; *DT =* 9.26) and those who did not smoke (*M =* 26.8; *DT =* 7.64), where those who said they smoked had a higher mean score.

Table 5 shows the significant differences in parents’ attitude toward drugs (*t = −*11.46; *p* < 0.001) depending on whether the adolescents’ parents allow them or would allow them to drink alcohol. Thus, those who said they are not or would not be allowed to drink had a higher mean score (*M =* 3.70; *DT =* 0.44) on parental concern about use than those who said they are (*M =* 3.40; *SD =* 0.44).

Furthermore, there were significant differences in adolescents’ attitude toward use (*t =* 5.33; *p* < 0.001) depending on whether their parents allowed them or would allow them to drink alcohol. Thus, those who said they were allowed or would be allowed to drink had a higher mean score on favorable attitude toward drug use (*M =* 29.12; *SD =* 8.44) than those who said they were not (*M =* 26.49; *SD =* 8.08).

Table 5 shows the significant differences in parents’ attitude toward drugs (*t* = −5.89; *p* < 0.001) in relation to whether the adolescents’ parents allow or would allow them to smoke. Thus, those who said they were allowed or would be allowed to smoke had a higher mean score (*M* = 3.34; *SD* = 0.52) on parental concern about use than those who said they were not (*M* = 6.63; *SD* = 0.44).

In addition, significant differences were detected in adolescents’ attitude toward drug use (*t* = 5.60; *p* < 0.001) depending on whether their parents allowed them or would allow them to drink alcohol. Thus, those who said they were not allowed to or would not be allowed to drink had a higher mean score on favorable attitude toward drug use (*M* = 26.91; *SD* = 8.15) than those who said they were allowed to (*M* = 31.29; *DT* = 9.06). That is, the adolescents who said their parents did not let them drink alcohol had a favorable attitude toward drug use.

### 3.4. Logistic Regression Model: Attitudes toward Use and Self-Efficacy in Refusing as Predictors of Drinking Alcohol

For the logistic regression analysis, we employed the dichotomous variable (no, yes) on drinking alcohol as the dependent variable. The predictors that entered in the equation were: favorable adolescent attitude toward drugs, parental concern about drug use, and self-efficacy in refusing to drink (Table 6).

The odds ratio, or cross-product ratio determined for each variable, shows that (a) the risk of drinking alcohol is higher in adolescents with a favorable attitude toward drug use, and (b) parental concern about drug use and self-efficacy in refusing under social pressure acted as protective factors in their likelihood of drinking alcohol. Thus, those participants who had higher mean scores in this dimension of self-efficacy in refusing to drink showed a lower risk of becoming drinkers.

Although the variables entered have explanatory capacity over the dependent variable (χ^2^ = 392.11; *df* = 5; *p* < 0.001), the model did not show satisfactory fit according to the Hosmer–Lemeshow test (χ^2^ = 23.04; *df* = 8; *p* = 0.003). The Nagelkerke R2 coefficient showed that 36.8% of the variability in the variable dependent is explained by the logistic regression model. Similarly, based on the case classification table, the likelihood that the logistic function is right is 72.7%, with a 0.27 false-positive rate and a 0.72 false-negative rate.

**Figure 1:**
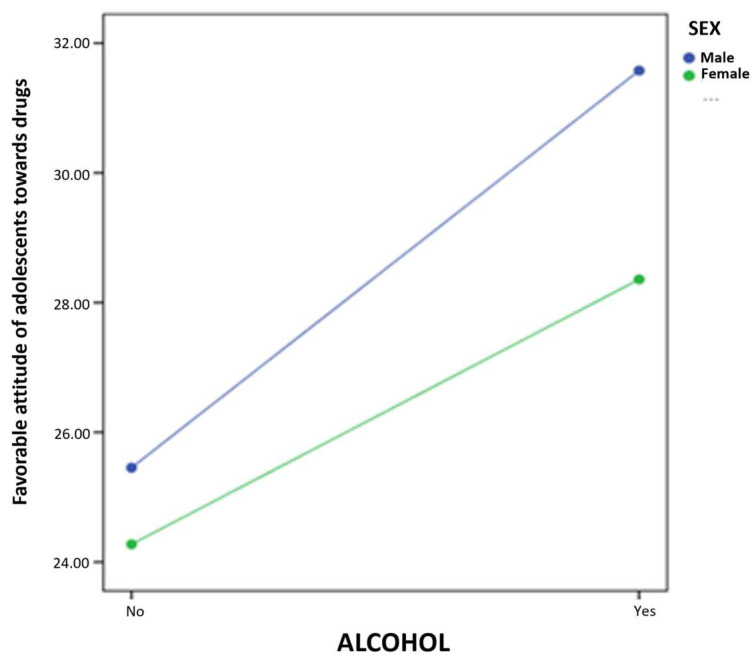
Interaction of alcohol use and sex in the prediction of favorable attitude toward drugs in adolescents.

The results of the simple moderation model report a statistically significant effect of alcohol consumption (B = 5.10, *p* < 0.001) on favorable attitude toward drugs. Furthermore, in this case, the coefficient of the interaction term is also significant (B = −2.03, *p* < 0.05). Using the pick-a-point approach, the prediction of alcohol consumption on favorable attitude toward drugs is calculated at different values of the moderator (male and female sex). This allows us to determine the conditional effect of the independent variable on the dependent variable at different values of the moderator. Thus, the results presented in Figure 1 suggest that the influence of the moderator variable occurs in both cases: male (B = 6.12, *p* < 0.001) and female (B = 4.08, *p* < 0.001).

### 3.5. Logistic Regression Model: Attitudes toward Use and Self-Efficacy in Refusing as Smoking Tobacco Predictors

In this case, smoking tobacco was entered in the logistic regression as the dichotomous dependent variable (no, yes). The same variables were entered in the logistic regression model for drinking alcohol as predictor variables. Self-efficacy in refusing to drink was retained because of the relationship in use of these two substances (Table 7).

The odds ratio for each variable shows that a) there is likelihood of lesser risk of being a smoker for adolescents who scored higher in self-efficacy of refusing under social pressure, or, in other words, this self-efficacy dimension would be acting as a protective factor against the likelihood of being a smoker, and b) a favorable attitude toward drugs is a risk factor for smoking tobacco. In smoking tobacco, the parental attitude (measured as concern about drug use) is not involved in the equation as a predictor.

Overall fit (χ^2^ = 183.867; *df* = 5; *p* < 0.001) was confirmed by the Hosmer–Lemeshow test (χ^2^ = 9.49; *gl* = 8; *p* = 0.302).

The Nagelkerke R2 shows that 23.3% of the variability in response was explained by the logistic regression model. Similarly, based on the case classification table, a probability of the logistic function being right is 83.5%, with a false-positive rate of 0.2 and a 0.17 false-negative rate.

**Figure 2:**
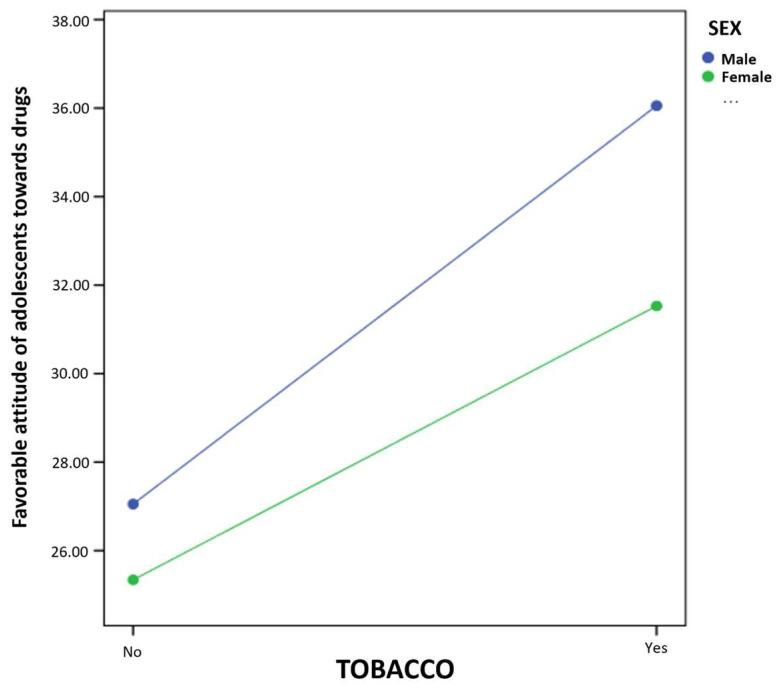
Interaction of tobacco use and sex in the prediction of favorable attitude toward drugs in adolescents.

Finally, the results of the simple moderation model report a statistically significant effect of tobacco consumption (B = 7.59, *p* < 0.001) on favorable attitudes toward drugs. Moreover, in this case, the coefficient of the interaction term is also significant (B = −2.81, *p* < 0.05). Using the pick-a-point approach, the prediction of smoking on favorable attitude toward drugs is calculated at different values of the moderator (male and female sex). Thus, the results presented in Figure 2 suggest that the influence of the moderator variable occurs in both cases: male (B = 9.00, *p* < 0.001) and female (B = 6.19, *p* < 0.001).

## 4. Discussion

Adolescent use of harmful substances has been increasing yearly, and the starting age has decreased considerably [25,72]. Our results showed that when the adolescents’ attitude toward drugs is favorable, self-efficacy decreases, so there is a higher percentage of users of these substances when the peer group, as a key point of reference, exerts strong pressure [21].

With regard to parental concern about use of drugs and self-efficacy toward refusing alcohol, the results showed that stronger family concern generates a higher level of self-efficacy in their children. Therefore, the family environment has a fundamental role insofar as the influence it exerts on the patterns of use of substances harmful to their health [20,21].

Concerning self-efficacy in refusing social pressure and frequency drinking alcohol, it was determined that adolescents’ drinking increases when they do not oppose the influence of social pressure. Then, attraction of dangerous situations when adolescents drink alcohol is a determinant since, combined with the influence the social setting has on the variables, it is a decisive element in adolescents starting to drink alcohol as well as for excessive drinking of alcohol [42]. Something similar takes place in favorable attitudes toward drugs and the assessment the adolescents make of their relationship with their parents, since the worse their relationship with their parents is, the more in favor of using drugs the children are. In fact, in some families, the parents think that experiences with alcohol are inevitable, and these parents sometimes drink too much [32]. Therefore, the quality of a parent/child relationship in which children are well informed before they start drinking alcohol can influence and tip their behavioral attitude [45,46,47,48]. The same is true of tobacco, as when the father, mother or both parents are smokers, they are exerting a direct influence on the adolescent with regard to normalizing situations in which such behavior is performed [49,50,51,52,53].

Continuing with the results of self-efficacy in refusing alcohol, the findings showed that male adolescents drank more frequently than female adolescents, even though their self-efficacy in refusing alcohol was higher. Similar studies have obtained results along this line, but in relation to smoking tobacco. Olivari et al. [54] showed that greater self-efficacy is related to lesser use of tobacco in adolescence, but only for girls. These results, together with the findings of the moderation models, may be related to the differential vulnerability between boys and girls to the risk of using legal and illegal drugs [73]. Thus, male adolescents seem to show more confidence in their own abilities, and therefore would believe that they can refuse more often than women who feel more vulnerable to perceived pressure [74], although consumption in women is lower. These findings point to new lines of study concentrated on the differential analysis of risk factors between the genders.

Nevertheless, significant gender differences in adolescent attitude toward drug use have been described, in which boys are more prone to drug use than girls are, and drink alcohol and use cannabis more than girls do [23]. The mother and father are determinant figures for their sons and daughters in drinking alcohol [21]. Thus, with regard to parents’ attitude toward drugs and adolescents’ attitude toward use, children whose parents brought them up with an indulgent style used drugs less [23]. Our results showed that parents’ attitude toward drugs is related to the mother, but not to the father. Similar studies have determined that the mother tends to value her relationship with her children more positively, and therefore has more parental knowledge than the father, knowing more about where the children are, their activities and their friends; here, control is associated with greater likelihood of not using legal or illegal drugs [75,76]. This communication and control of adolescents can become an effective strategy for helping young people to develop and maintain a sensible relationship with legal or illegal drug use [74].

Another study conducted by Jacob et al. [32] suggested that the role of the family is critical in educating adolescents in tolerance of drinking alcohol and as the basis for acceptance of this social norm across the general population. Continuing with the role of the family, parenting styles also have been shown to be influential in adolescent substance use, and other risk behavior as violence [77]. Some studies have determined that an authoritarian parenting style in which parents try to mold, control and evaluate their children’s behavior based on a set of absolutely strict rules and punishment protects against substance use by adolescents. However, the permissive style which is warmer and grants more autonomy is associated with higher rates of legal and illegal drug use by adolescents [78]. This is why in the future it would be of interest to continue examining the role of families in adolescent drug use, including parenting styles and evaluation of parental attitudes in protecting against use of alcohol and tobacco.

It was also confirmed that family support is decisive for preventive intervention, not only within the parental environment, but in the social environment. Even in a normative context, it is a crucial aspect for shifting cultural norms and producing a change in the behavior of youth in the population they belong to [32].

Moreover, adolescents who show more self-efficacy in refusing drug use are those with the lowest risk of becoming users, and include parental attitude or their concern about drug use in their explanatory model of drinking alcohol. Thus, the attitude of parents is related to drinking alcohol, in line with other studies [20,21,38], while concern for smoking tobacco is not involved in the explanatory model of using this drug. In the future, we could analyze these differences in the influence of parental attitude, which might be due to a different focus of families on the immediate consequences of drinking alcohol compared to smoking tobacco, which would show its effects in the longer term.

Some limitations of this study should also be mentioned. Other variables that could affect and/or complement the results, such as parenting style which influences personality variables such as self-esteem, resilience, self-control, etc. as they relate to adolescent behavior with regard to substance use, were not included. The family model was not considered either. Adequate family functioning has been established as a factor that protects adolescents against drinking alcohol and smoking tobacco [79], so future studies should consider this in their analysis of this type of behavior. Neither can we overlook that the instruments applied were self-reports, so, although they had good reliability and validity, the answers of the adolescents may have been mediated by social desirability. With regard to the instruments of the study, it should also be mentioned that adolescents were asked about using “drugs” in a wider sense, without specifying whether legal, illegal or both. This could affect the students’ interpretation, and therefore their answers. Future studies should emphasize the specific substance being evaluated. We should also add that, although based on a representative sample of secondary school students, as all of them were from the province of Almeria, the conclusions may not be completely valid for the whole Spanish adolescent population. We should also mention that the reliability determined for the Parental Attitudes toward Drug Use Scale was low. Therefore, the results should be interpreted with caution, since these measurements might not be completely stable.

In our study, the sample was limited specifically to high school students, so the results may only be taken into consideration in this concrete population group and no other. Therefore, future studies should include other variables and widen the age of the sample, and add some questions on use of drugs other than alcohol and tobacco.

## 5. Conclusions

Use of harmful substances is an established reality in adolescence. However, young people are beginning such activities at a considerably earlier age, and therefore, in-depth research must be undertaken to be able to respond to the needs of today’s society.

We have observed that self-efficacy for refusing drugs is a fundamental element in avoiding drinking alcohol and smoking tobacco, where parental attitude or their concern for drug use is present as an explanatory factor and is therefore related to the outcome. Specifically, self-efficacy in refusing social pressure must not be overlooked, and there is still much work to be done, as we have shown above that the amount of drinking alcohol increases when the adolescent does not oppose the influence of social pressure.

From this perspective, acquiring information on self-efficacy in refusing alcohol and the attitude adolescents have toward drugs as well as their perception of their parents contribute data for planning future intervention programs that reduce the harmful impact these practices have on the health of youths.

## Figures and Tables

**Table 1:** Sociodemographic characteristics of the sample.

Variables		Male	Female	Total
Age	*M*	15.11	15.12	16.54
	*SD*	0.91	0.94	1.48
			%	*n*
Sex	Males		43	172
	Female		57	228
Grade (Academic year)	3°		55	707
	4°		45	577

*M* = Mean; *SD* = Standart Desviation.

**Table 2:** Correlation analysis of variables. Pearson’s correlations (*N* = 1287).

		Social Pressure Refusal Self-Efficacy	Opportunistic Refusal Self-Efficacy	Emotional Relief Refusal Self-Efficacy	Favorable Attitude of Adolescents Toward Drugs	Parental Concern about Drug Use
Favorable attitude of adolescents toward drugs	Pearson’s correlation	−0.333 **	−0.254 **	−0.294 **		
	Sig. (bilateral)	0.000	0.000	0.000		
Parental concern about drug use	Pearson’s correlation	0.199 **	0.164 **	0.171 **	−0.278 **	
	Sig. (bilateral)	0.000	0.000	0.000	0.000	
Relationship with father/guardian	Pearson’s correlation	0.036	−0.008	0.030	−0.066 *	0.025
	Sig. (bilateral)	0.196	0.780	0.288	0.019	0.362
Relationship with mother/guardian	Pearson’s correlation	0.035	0.002	0.034	−0.109 **	0.066 *
	Sig. (bilateral)	0.208	0.932	0.222	0.000	0.017
Alcohol drinking frequency	Pearson’s correlation	−0.170 **	−0.051	−0.091 **	0.215 **	−0.133 **
	Sig. (bilateral)	0.000	0.067	0.001	0.000	0.000
Smoking frequency	Pearson’s correlation	−0.004	−0.030	−0.024	0.095 **	0.108 **
	Sig. (bilateral)	0.898	0.286	0.395	0.001	0.000

* *p* < 0.05; ** *p* < 0.01.

**Table 3:** Self-efficacy in refusing alcohol, parents’ attitude toward drugs and favorable attitude toward drugs. Descriptive statistics and t-test by gender.

	Gender						*t*	*p*	*d*
	Boys			Girls					
	*N*	*M*	*SD*	*N*	*M*	*SD*			
Social pressure refusal self-efficacy	606	22.33	6.93	681	21.43	6.91	2.34 *	0.019	0.13
Opportunistic refusal self-efficacy	606	36.25	9.62	681	38.93	7.49	−5.51 ***	0.000	−0.31
Emotional relief refusal self-efficacy	606	35.39	9.71	681	37.52	8.07	−4.25 ***	0.000	−0.24
Parental concern about drug use	606	3.57	0.49	681	3.64	0.43	−2.66 **	0.008	−0.15
Favorable attitude of adolescents toward drugs	606	28.29	8,74	681	26.58	7.90	3.67 ***	0.000	0.21

* *p* < 0.05; ** *p* < 0.01; *** *p* < 0.001.

**Table 4:** Self-efficacy in refusing alcohol and attitudes toward drugs. Student’s t-test depending on consumption of alcohol or its absence (no/yes).

Drink alcohol			**Social Pressure Refusal Self-Efficacy**	**Opportunistic Refusal Self-Efficacy**	**Emotional Relief Refusal Self-Efficacy**	**Parents’ Attitude toward Drugs**	**Favorable Attitude of Adolescents toward Drugs**
	No	*N*	620	620	620	620	620
		*M*	25.34	38.57	38.13	3.70	24.89
		*SD*	5.87	7.95	7.93	0.43	7.32
	Yes	*N*	662	662	662	662	662
		*M*	18.58	36.81	35.00	3.51	24.89
		*SD*	6.23	9.22	9.56	0.47	7.32
	*t*		19.99 ***	3.65 ***	6.37 ***	7.52 ***	−10.83 ***
	*p*		0.000	0.000	0.000	0.000	0.000
	*d*		1.08	0.20	0.36	0.36	0.61

*** *p* < 0.001.

**Table 5:** Parents’ and adolescents’ attitudes toward drugs. Student’s t-test depending on whether they smoke or not (no/yes), permissiveness of parents toward drinking alcohol (no/yes) and smoking (no/yes).

			Parents’ Attitude Toward Drugs	Favorable Attitude of Adolescents Toward Drugs
Smoke	No	*N*	1060	1060
		*M*	3.63	26.8
		*SD*	0.45	7.64
	Yes	*N*	218	218
		*M*	3.46	33,21
		*SD*	0.49	9.26
	*t*		4.78 ***	−10.49 ***
	*p*		0.000	0.000
	*d*		0.36	
If you drink alcohol or would like to do so, do your parents or would they allow you to?	No	*N*	844	844
		*M*	3.70	26.49
		*SD*	0.44	8.08
	Yes	*N*	417	417
		*M*	3.40	29.12
		*SD*	0.44	8.44
	*t*		−11.46 ***	5.33 ***
	*p*		0.000	0.000
	*d*		−0.68	
If you smoke or would like to smoke, do your parents or would they allow you to?	No	*N*	1131	1131
		*M*	3.63	26.91
		*SD*	0.44	8.15
	Yes	*N*	124	124
		*M*	3.34	31.29
		*SD*	0.52	9.06
	*t*		−5.89 ***	5.60
	*p*		0.000	0.000
	*d*			

*** *p* < 0.001.

**Table 6:** Results of the logistic regression analysis for likelihood of drinking alcohol.

Variables	β	Std. Error	Wald	df	Sig.	Exp(β)	CI 95%
Parental concern about use	−0.545	0.160	11.613	1	0.001	0.580	0.424, 0.793
Favorable attitude of adolescents toward drugs	0.049	0.009	29.502	1	0.000	1.050	1.032, 1.069
Social pressure refusal self-efficacy	−0.189	0.014	179.154	1	0.000	0.828	0.805, 0.851
Opportunistic refusal self-efficacy	0.038	0.017	5.051	1	0.025	1.038	1.005, 1.073
Emotional relief refusal self-efficacy	0.018	0.018	1.011	1	0.315	1.018	0.983, 1.054
Constant	2.838	0.756	14.089	1	0.000	17.082	

**Table 7:** Results derived from the logistic regression for likelihood of smoking tobacco.

Variables	β	Std. Error	Wald	df	Sig.	Exp(β)	CI 95%
Parental concern about use	−0.224	0.165	1.841	1	0.175	0.799	0.578, 1.105
Favorable attitude of adolescents toward drugs	0.082	0.010	61.267	1	0.000	1.086	1.063, 1.108
Social pressure refusal self-efficacy	−0.091	0.014	43.843	1	0.000	0.913	0.889, 0.938
Opportunistic refusal self-efficacy	0.052	0.018	8.689	1	0.003	1.053	1.018, 1.090
Emotional relief refusal self-efficacy	−0.025	0.018	1.954	1	0.162	0.976	0.943, 1.010
Constant	−2.397	0.823	8.479	1	0.004	0.091	

## Data Availability

The data presented in this study are available on request from the corresponding author.
